# Reweighting of Binaural Localization Cues Induced by Lateralization Training

**DOI:** 10.1007/s10162-021-00800-8

**Published:** 2021-05-06

**Authors:** Maike Klingel, Norbert Kopčo, Bernhard Laback

**Affiliations:** 1 Acoustics Research Institute, Austrian Academy of Sciences, 1040 Vienna, Austria; 2 Department of Cognition, Emotion, and Methods in Psychology, Faculty of Psychology, University of Vienna, 1010 Vienna, Austria; 3 Institute of Computer Science, Faculty of Science, P. J. Šafárik University in Košice, 04180 Košice, Slovakia

**Keywords:** Spatial hearing, Interaural time difference, Interaural level difference, Plasticity, Adaptation, Trading ratio

## Abstract

Normal-hearing listeners adapt to alterations in sound localization cues. This adaptation can result from the establishment of a new spatial map of the altered cues or from a stronger relative weighting of unaltered compared to altered cues. Such reweighting has been shown for monaural vs. binaural cues. However, studies attempting to reweight the two binaural cues, interaural differences in time (ITD) and level (ILD), yielded inconclusive results. This study investigated whether binaural-cue reweighting can be induced by lateralization training in a virtual audio-visual environment. Twenty normal-hearing participants, divided into two groups, completed the experiment consisting of 7 days of lateralization training, preceded and followed by a test measuring the binaural-cue weights. Participants’ task was to lateralize 500-ms bandpass-filtered (2–4 kHz) noise bursts containing various combinations of spatially consistent and inconsistent binaural cues. During training, additional visual cues reinforced the azimuth corresponding to ITDs in one group and ILDs in the other group and the azimuthal ranges of the binaural cues were manipulated group-specifically. Both groups showed a significant increase of the reinforced-cue weight from pre- to posttest, suggesting that participants reweighted the binaural cues in the expected direction. This reweighting occurred within the first training session. The results are relevant as binaural-cue reweighting likely occurs when normal-hearing listeners adapt to new acoustic environments. Reweighting might also be a factor underlying the low contribution of ITDs to sound localization of cochlear-implant listeners as they typically do not experience reliable ITD cues with clinical devices.

## INTRODUCTION

Spatial hearing mechanisms allow us to determine the location of a sound source and are important for understanding speech in complex environments as well as orienting in space. For vertical-plane localization, we rely primarily on monaural spectral-shape cues, while for azimuthal sound localization the binaural cues of interaural time difference (ITD) and interaural level difference (ILD) are most important (for a review on localization cues, see, e.g., Middlebrooks and Green 1991). This study investigates the flexibility with which the two binaural cues are integrated to form an azimuthal percept.

The extent to which each binaural cue contributes to azimuthal sound localization mainly depends on the frequency content of the sound. ITDs are dominant at low frequencies whereas ILDs are dominant at higher frequencies (Macpherson and Middlebrooks 2002; Strutt 1907; Wightman and Kistler 1992). The relative weight with which ITDs and ILDs contribute to a spatial percept has traditionally been measured using ITD/ILD trading ratios, estimated by presenting one cue at a fixed value and instructing the participants to adjust the other cue until the sound is perceived centrally (Durlach and Colburn 1978).

While trading ratios depend primarily on the frequency content of the stimulus, they are also influenced by other stimulus factors such as overall intensity (David et al. 1959; Deatherage and Hirsh 1959) or the inter-click interval of click trains (Stecker 2010). Contextual or environmental factors also influence the ITD/ILD weighting. Rakerd and Hartmann (2010) observed that in reverberant environments, participants’ responses follow ILDs while localizing 500 Hz sine tones, a stimulus for which ITDs are dominant in anechoic environments. Moreover, ITD/ILD trading ratios depend on which cue is adjusted. Namely, the to-be-adjusted cue receives greater weight, presumably because attention is shifted toward it (Lang and Buchner 2008) or due to cue-specific adaptation (Moore et al. 2020).

Trading ratios are usually only measured at a single point in time. However, the relationship of sound localization cues to corresponding locations in space may change during one’s life. Therefore, adaptation to altered spatial cues has been extensively studied (see Carlile 2014, and King et al. 2011, for reviews), highlighting the plasticity of the auditory system. Observed adaptation can either result from the establishment of a new spatial map of the altered cues (i.e., a modified relationship between sound localization cues and corresponding locations in space; Keating et al. 2015; Knudsen 2002; Shinn-Cunningham et al. 1998; Trapeau and Schönwiesner 2015) or from a stronger relative weighting of unaltered compared to altered spatial cues, referred to as reweighting (Keating et al. 2013; Kumpik et al. 2010; van Wanrooij and van Opstal 2007). In these latter studies, participants learned to increase the relative weight of monaural compared to binaural cues for azimuthal sound localization.

The current study examines whether not only monaural vs. binaural but also the two binaural cues ITD and ILD can be reweighted. Only two previous studies explicitly attempted to achieve binaural-cue reweighting, yielding inconclusive results. Jeffress and McFadden (1971) used a left/right discrimination task with ITDs and ILDs favoring opposite ears and presented feedback consistent with only one of the cues but did not find any changes in the ITD/ILD weighting after training. Potential reasons for their null result are that (1) the training regimen (i.e., left/right discrimination) was not sufficiently intuitive, (2) the stimuli were noise bands centered at 500 Hz and thus in a frequency range where only ITDs but not ILDs arise naturally (except for sound sources near the head), (3) the binaural cues used were not sufficiently salient as they were close to the binaural-cue threshold, and (4) auditory and visual stimuli were not presented simultaneously, preventing bottom-up multisensory integration that may be required to induce reweighting. Kumpik et al. (2019) presented auditory stimuli with either randomized ITDs (and stable ILDs) or randomized ILDs (and stable ITDs) while participants completed a visual oddball task. They observed an increase in ILD weighting after ITDs were randomized, but no increase in ITD weighting after ILDs were randomized. Since the auditory stimuli were task irrelevant, they might not have received enough attention to induce an effect in both directions. Additionally, the potential to increase the ITD weighting may have been restricted due to applying reverberation, presumably making the ITDs less reliable. This is in line with Rakerd and Hartmann’s (2010) observation that responses follow ILDs in reverberant environments. Furthermore, Kumpik et al. (2019) found an even stronger increase in ILD weighting for a condition in which no cue was randomized (i.e., spatially consistent ITDs and ILDs were presented), making it difficult to draw strong conclusions. Considering the changing relative contributions of ITD and ILD depending on stimulus and contextual factors as discussed above, binaural-cue reweighting likely plays a role in adapting to variable acoustic environments. Therefore, these inconclusive results are rather surprising and worthy of further consideration.

**Fig. 1:**
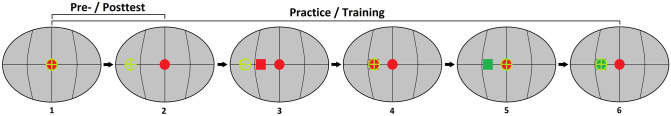
Time course of a trial during testing (panels 1–2), practice (panels 1–6), and training (panels 1–6). (1) Participants oriented towards the reference position straight ahead (indicated by a red sphere) by turning their head (guiding a green crosshair) toward it and pressed a button to elicit the sound presentation. (2) Participants turned their head to the perceived azimuth and pressed a button at the end of the head turn to record the response azimuth (in this example, they performed a head-turn to the left). During testing, the next trial was initiated after the button press while during practice and training, the trial continued with steps 3–6. (3) Visual reinforcement (a rotating red cube) appeared at the reinforced-cue azimuth. (4) The participants confirmed the reinforced-cue azimuth via a head turn to the reinforced-cue azimuth and a button press. (5) The visual reinforcement turned green, and participants returned to the reference position. After another button press, the auditory stimulus was presented again while the visual reinforcement was still visible. (6) Participants performed another head turn to the reinforced-cue azimuth and pressed the button. In steps 4 and 6, the button-press was accepted only if the head-orientation (green crosshair) was within ± 5° of the reinforced-cue azimuth

Here, we reexamined the question of whether the auditory system can adjust the binaural-cue weights via training. We trained participants using a lateralization task in a well-controlled virtual environment involving auditory, visual, and proprioceptive information based on the procedure used in Majdak et al. (2013). A schematic illustration of the visual environment and the task is shown in Fig. 1. The training used two forms of visually guided auditory spatial calibration: (1) visual stimuli presented after the auditory stimuli to serve as top-down feedback (see red square in panel 3 in Fig. 1) comparable to the “feedback” experiments of Shinn-Cunningham et al. (1998), and (2) simultaneously presented auditory and visual stimuli to encourage multisensory bottom-up processes (see panel 5 in Fig. 1) equivalent to those evoked in the ventriloquism aftereffect paradigm (Reccanzone 1998). In contrast to Kumpik’s et al. (2019) study, the auditory stimuli were critical to perform the task and unlike Jeffress and McFadden (1971), we used a stimulus spectrally focused at an intermediate frequency region, to ensure that neither ITDs nor ILDs are used by default. Since the sensitivity to binaural cues can be modified based on the statistics of the sound (Dahmen et al. 2010), we additionally manipulated the across-trial stability of the two binaural cues during training by varying one of the cues over a larger range than the other. Finally, a variety of combinations of ITDs and ILDs were presented to facilitate generalization of reweighting across spatial configurations and to minimize chances for strategic responding (i.e., memorizing specific stimuli or azimuths and responding accordingly).

## METHODS

### Participants

Twenty-six normal-hearing adult participants took part in the study. All participants gave informed consent before participating and received monetary compensation for their participation. Basic lateralization ability as well as sensitivity to both ITDs and ILDs were assessed in a practice session (see “Procedure” for details) and served as an inclusion criterion. Of these 26 participants, three had to be excluded because they experienced dizziness induced by the head-mounted display used for the presentation of the visual environment. Another two participants exercised their right to terminate the experiment prematurely and were therefore excluded. In addition, one participant was excluded due to poor ITD sensitivity (i.e., the percentage of responses at the correct side in practice trials with ILDs fixed at zero was at chance level). Twenty participants (10 female, mean age 26.9 years, *SD* = 4.13, range 21–40 years) therefore completed the experiment. We used two experimental groups: ITDs were reinforced for the ITD group and ILDs were reinforced for the ILD group. Participants were pseudo-randomly assigned to one of the groups, ensuring balanced age, sex, and basic lateralization ability. Ten participants (five female, mean age: 27 years, *SD* = 5.27), were assigned to the ITD group. The other ten participants (five female, mean age: 26.8 years, *SD* = 2.86), were assigned to the ILD group. The research protocol was submitted to the acoustics research institute's ethics committee for consideration, comments, guidance, and approval. After taking ethical issues, local laws and regulations into account, the ethical committee approved the protocol.

### Apparatus and Stimuli

Participants stood on a platform surrounded by a circular railing inside a sound booth. The experiment was controlled by custom-written software routines that were run on two communicating personal computers (via UDP protocol), that shared the tasks of acquiring head tracking data, creating and presenting acoustic stimuli, and rendering the visual environment. Binaural auditory stimuli were generated using a computer and output via a digital audio interface (ADI-8, RME) at a 96-kHz sampling rate and presented via headphones (HD 580, Sennheiser). Visual stimuli were presented binocularly via a head-mounted display (Oculus Rift DK1). Participants’ head position and orientation were tracked with a head-mounted tracking sensor (Flock of Birds, Ascension), and the visual environment was rendered accordingly in real time (the latency between head movements and the updated visual information was less than 37.3 ms). The rendering of the visual environment using Pure Data (with GEM, IEM, Graz) was based on the left/right rotation information from the tracking sensor while the up/down information was ignored to force participants to respond only in the horizontal plane. The virtual visual environment consisted of a reference position straight ahead, a crosshair in the direction of the head orientation, a single horizontal line at eye level, and vertical lines every 15° in azimuth for guidance (Fig. 1).

**Fig. 2:**
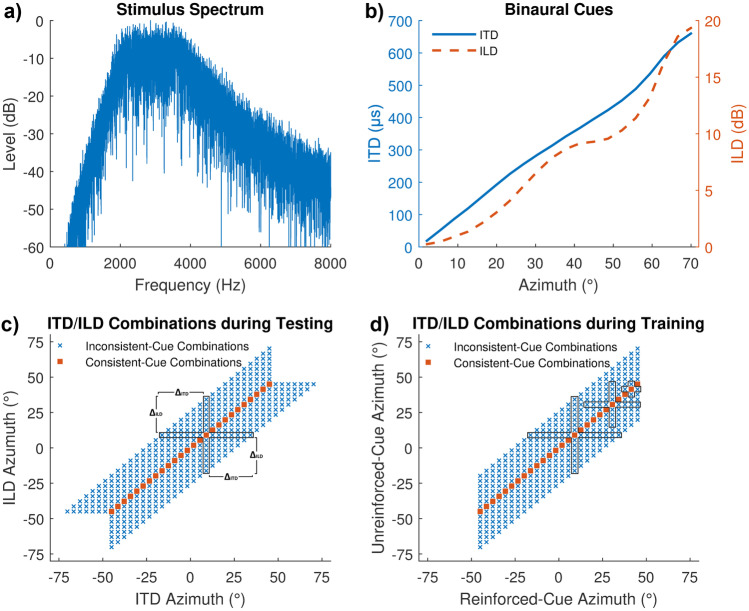
Experimental setup and stimuli. **a** Spectrum of the auditory stimuli used. **b** Functional relation between the azimuth and the binaural cues as derived by Xie (2013) from the KEMAR head-related impulse responses (HRIRs), with ITDs (solid line) referring to the left ordinate and ILDs (dashed line) referring to the right ordinate. ITDs are based on broadband cross-correlation of the left and right ear HRIRs. ILDs are based on HRTF magnitudes at 2.8 kHz. **c** ITD/ILD-azimuth combinations used in the pre- and posttest. The frame indicates the azimuthal offsets Δ_ITD_ and Δ_ILD_ that were used to estimate the parameters of the regression analysis for the pre-/posttest data at one example azimuth (9°). **d** All cue combinations used in the training. For the ITD group, reinforced and unreinforced cues were ITD and ILD, respectively, and for the ILD group, reinforced and unreinforced cues were ILD and ITD, respectively. The frames indicate the data that were used to estimate the parameters of the regression analysis for the training data at example azimuths of 9° (large frame), 30.6° (medium frame), and 41.4° (small frame). The reduction of frame size towards the edge was required to ensure symmetric distributions (see text)

Auditory source stimuli were white noise bursts, randomly generated on each trial, which were filtered with a 2–4 kHz^1^ Butterworth band-pass filter (roll-off outside the passband: approximately 30 dB/oct; Fig. 2a), on which ITDs ranging from −662 to +662 µs and ILDs ranging from −19.4 to +19.4 dB were imposed. These cues correspond to an azimuthal range from −70.2° to +70.2° (Fig. 2b), as estimated by Xie (2013) using the head-related transfer functions (HRTFs) of the KEMAR head with DB-61 small pinna at a source distance of 1.4 m. In Xie (2013), the ITD values were obtained via broadband cross-correlation of the left and right ear head-related impulse responses (HRIRs) and the ILD values were based on the HRTF magnitudes at 2.8 kHz (i.e., the center frequency of the auditory stimuli used in the present study). The stimuli were not HRTF filtered to ensure that they did not convey monaural spectral localization cues that are potentially informative about the azimuth of the stimulus, to reveal purely binaural-cue reweighting. The choice of the stimulus center frequency of 2.8 kHz was guided by the requirement of monotonically increasing ILDs with increasing azimuth at the center frequency within the desired stimulus azimuthal range of ± 70.2° to avoid ambiguous ILDs which would occur if the same cue value corresponded to multiple azimuths. Additionally, the chosen frequency range of 2–4 kHz lies in between frequency ranges that are typically either ITD- or ILD-dominant. It was therefore assumed that neither of the two binaural cues would be weighted particularly strongly and each binaural cue weight would thus have the potential to be increased. For frequencies above 1.4 kHz, thresholds for ITDs conveyed by the carrier signal (the so-called temporal fine structure) become unmeasurable (Brughera et al. 2013), and therefore, ITD cues are conveyed only via the stimulus’ temporal envelope. Bernstein and Trahiotis (1982), however, showed that low-frequency residual energy far below the nominal pass band of a stimulus can provide salient ITD cues, even if those cues are transmitted at a low sensation level. We therefore cannot rule out some contribution of fine-structure ITD cues. Note that the particular contribution of envelope or fine-structure ITD cues was not the focus of this study. The stimulus duration was 500 ms, including 50-ms raised-cosine on/off ramps. The mean overall sound pressure level (SPL) across ears was 65 dB. To discourage participants from using differences in the absolute level rather than ILDs for lateralization, the overall level was randomly roved from trial to trial within a ± 2.5 dB range.

Our study included stimuli with spatially consistent or inconsistent binaural cues. For consistent-cue conditions, the ITD and ILD cues corresponded to the same azimuth (squares in Fig. 2c, d), whereas for inconsistent-cue conditions, the ITD and ILD cues corresponded to disparate azimuths (“x” symbols in Fig. 2c, d). In the training sessions, 26 reinforced-cue azimuths (i.e., azimuths corresponding to the visually reinforced binaural cue) were distributed between −45° (left) and +45° (right) with a spacing of 3.6° (*x*-axis in Fig. 2d). This range corresponds to the field of view of the head-mounted display. In conditions with inconsistent ITD/ILD-combinations, the unreinforced-cue azimuths (i.e., azimuths corresponding to the unreinforced cue) were uniformly distributed ± 25.2° around each reinforced-cue azimuth (columns in Fig. 2d), also with an azimuthal spacing of 3.6°, resulting in a total range of unreinforced-cue azimuths from −70.2° to +70.2°. The disparity between ITD and ILD azimuths was intentionally limited (maximum of 25.2°) with the goal of avoiding the perception of split images (e.g., Hafter and Jeffress 1968). Based on Gaik’s (1993) results, we did not expect split-image perception to occur for the frequency range and binaural cue disparities used in this study. In the pre- and posttest, in which no cue was reinforced, all combinations of ITD and ILD azimuths used for either group during training were included (Fig. 2c). The rationale behind using so many different spatial configurations of ITD and ILD was to prevent the possibility of identifying individual stimulus azimuths and then responding strategically.

### Procedure

The general task involved indicating the perceived azimuth of an intracranial sound source via head turn. Because stimuli were relatively narrowband and contained no HRTF filtering, they were likely not externalized by the participants. Thus, participants had to map the perceived intracranial image to the visual response field. Moore et al. (2020) argue that head-pointing in a virtual audio-visual environment can be used to obtain reliable in-head lateralization judgments without training participants to externalize the auditory stimuli. The paradigm for estimating binaural cue weights was based on Stecker’s (2010) “open loop” and Macpherson and Middlebrooks’ (2002) methods. Namely, by asking participants to lateralize stimuli containing spatially inconsistent ITD and ILD, we inferred how much each cue contributed to the azimuthal percept by comparing the response azimuth to the azimuths of the binaural cues (the measure of interest). This method is not subject to the bias of traditional trading ratio measurements (e.g., Deatherage and Hirsh 1959), because no cue is actively manipulated by the participants (see Lang and Buchner 2008) and single stimuli are presented (see Moore et al. 2020).

The experimental phases are shown in Table 1. The experiment started with a *practice session* to get participants accustomed to the task and to check their lateralization ability and sensitivity to both binaural cues. It continued with a *pretest* to measure the initial ITD and ILD weights, a 7-day *training* (completed within a 2-week interval) in which one of the cues was reinforced, and a *posttest* to remeasure the weights after the training. The approximate duration of the sessions was 2 h on the first day and 1.5 h per day on days 2 to 7. The choice of the number of training sessions and their duration was guided by the training regimen used in Kumpik et al. (2010).

**Table 1:** Time course (top to bottom and left to right) of experimental phases. On day 1, participants completed the practice session, followed by the pretest, followed by half a training session. On days 2–6, participants completed one training session each. On day 7, participants completed half a training session followed by the posttest. The seven testing sessions were completed within a 2-week interval

Day 1	Day 2	Day 3	Day 4	Day 5	Day 6	Day 7
Practice (130 trials)						
Pretest (446 trials)						
Training (195 trials)	Training (390 trials)	Training (390 trials)	Training (390 trials)	Training (390 trials)	Training (390 trials)	Training (195 trials)
						Posttest (446 trials)

**Practice.** The practice session (performed at the beginning of the experiment) consisted of 130 trials that differed from training trials (see training procedure below) only in the stimuli they used. The practice stimuli had either consistent ITD/ILD combinations (to measure basic lateralization ability) or they had either the ITD or the ILD fixed at zero while the other cue corresponded to a reinforced-cue azimuth (to test for sensitivity to the cues in isolation). Of the 130 practice trials, 78 (3 per reinforced-cue azimuth) contained consistent-cue stimuli, 26 (each reinforced-cue azimuth once) contained stimuli with ILD fixed at zero, and 26 (again each reinforced-cue azimuth once) contained stimuli with ITD fixed at zero. The trials were presented in random order while ensuring that the first 26 trials were consistent-cue combinations. Visual reinforcement was provided in each trial, as in the training (described below).

**Testing.** The pretest and the posttest were identical for the two groups and did not include visual reinforcement (see steps 1 and 2 of Fig. 1). On each trial, participants listened to the auditory stimulus while facing straight ahead (the reference position) and then indicated the perceived azimuth of the auditory stimulus via head turn and a button press (i.e., the azimuth indicated by the head turn at the time of the button press was recorded as the response azimuth). When they returned to the reference position, the session continued with the next trial. A total of 446 trials were presented, namely each ITD/ILD combination shown in Fig. 2c was presented once. These comprised all ITD/ILD combinations included in the training phase for both groups. The trials were presented in random order and after each 150 trials, participants took a short break.

**Training.** For training, we used a lateralization procedure based on the procedure used in Majdak et al. (2013). We adapted it by restricting it to the horizontal (azimuthal) dimension and by using the head-pointing technique. The training procedure consisted of 6 steps (shown in Fig. 1): (1) listening to the auditory stimulus while at the reference position straight ahead (initiated by pressing the button at the reference position), (2) indicating the perceived azimuth via head turn and button press, (3) receiving visual reinforcement (a rotating cube) at the reinforced-cue azimuth, (4) finding and confirming the reinforced-cue azimuth via head turn and button press, (5) returning to the reference position and listening to the same auditory stimulus again (initiated with a button press) while the visual reinforcement is still visible, and (6) confirming the reinforced-cue azimuth again via head turn and button press after which the visual reinforcement disappears. Participants were instructed to remain at the reference position for the duration of the auditory stimulus (500 ms) and reminded to do so throughout the experiment. However, participants were not physically prevented from initiating head turns during sound presentation. Nevertheless, even if they did start to turn their heads before the stimulus ended, there is no reason to expect that this would confound the experimental variables under investigation. After every 65 training trials, participants took a short break.

Auditory stimuli included both inconsistent and consistent ITD/ILD-combinations, as shown in Fig. 2d. The training procedure was the same for the two groups except for which cue was visually reinforced and presented in a limited azimuth range. Thus, for the ITD group, ITD azimuths did not exceed ± 45° and for the ILD group, ILD azimuths did not exceed ± 45°. This ensured that the visual reinforcement was visible while at the reference position, as ± 45° was the field of view of the head-mounted display. A full training session consisted of 390 trials presented in random order. On days 1 and 7, participants completed only half sessions (195 trials each, created by splitting a randomized item list for a full session) to prevent fatigue because the pre- and posttest were also completed on these days. On days 2 to 6, participants completed full training sessions.

### Analysis

The data were analyzed using MATLAB R2018b (The MathWorks, Natick, MA). Statistical analyses of results were performed using SPSS Statistics 20 (IBM, Armonk, NY). We estimated pre- and posttest binaural cue weights separately for each participant based on a regression analysis fitted separately for each azimuth α (between 1.8° and 45° with a 3.6° spacing between azimuths) after mirroring the data across the midline to get more reliable estimates (assuming left/right symmetry). The regression model equation is as follows:

$${R}_{ITD}\left(\alpha , {\Delta }_{ITD}\right)={k}_{ITD}\left(\alpha \right)*{\Delta }_{ITD}+{Q}_{ITD}\left(\alpha \right)$$

$${R}_{ILD}\left(\alpha , {\Delta }_{ILD}\right)={k}_{ILD}\left(\alpha \right)*{\Delta }_{ILD}+{Q}_{ILD}\left(\alpha \right)$$

$${w}_{ILD}\left(\alpha \right)=\frac{\mathit{atan}\left(\frac{{k}_{ILD}\left(\alpha \right)}{{k}_{ITD}\left(\alpha \right)}\right)}{\frac{\pi }{2}}$$

1 $$Q\left(\alpha \right)=\frac{{Q}_{ILD}\left(\alpha \right)+{Q}_{ITD}\left(\alpha \right)}{2}$$

where *R*_*ITD*_ (*R*_*ILD*_) is the participant’s response azimuth in a trial with ITD (ILD) corresponding to the azimuth *α* + *Δ*_*ITD*_ (*α* + *Δ*_*ILD*_) and ILD (ITD) corresponding to *α*. The parameters *k*_*ITD*_ and *k*_*ILD*_ are the estimated linear regression slopes at azimuth α (determining the individual binaural cue weight contributions), and *Q* is the estimated response azimuth for consistent cues corresponding to azimuth α. Parameter *k*_*ITD*_ (*k*_*ILD*_) was estimated at each azimuth by considering various azimuthal offsets (from -25.2° to +25.2° in 3.6° steps) of the cue, *Δ*_*ITD*_ (*Δ*_*ILD*_), while setting the offset of the other cue, *Δ*_*ILD*_ (*Δ*_*ITD*_), to zero. Thus, referring to Fig. 2c, the model was fitted for each azimuth *α*, indicated by a square, by considering only items of the row (for *k*_*ITD*_) and column (for *k*_*ILD*_) that included that square (an example set of data used for the estimation of parameters at *α* of 9° is indicated by the black frame in Fig. 2c). These estimates of *k*_*ITD*_ and *k*_*ILD*_, representing orthogonal vectors, were then combined to derive the ILD weight ${w}_{ILD}$ (note that ${w}_{ITD}$ = 1 – ${w}_{ILD}$). Finally, parameter *Q* was estimated as the average of the constants obtained in the regressions for ITD and ILD.

In addition to the estimation of pre- and posttest binaural cue weights, we sought to determine binaural-cue weights across the course of the training. This required, however, a modification of the procedure. The pre- and posttest data fulfilled the requirement of the regression analysis of a balanced distribution (specifically ± 25.2°) of ITD azimuths around ILD azimuths and vice versa. During training, this balanced distribution was available only for azimuths up to 19.8°, as reinforced-cue azimuths were limited to ± 45°. When analyzing the training data, we therefore gradually limited the range of *Δ*_*ITD*_ and *Δ*_*ILD*_ with increasing azimuth for azimuths more lateral than 19.8°, always ensuring a balanced, albeit reduced distribution (for example, at 41.4°, only the two neighboring azimuths as well as the consistent-cue condition were considered, see Fig. 2d). For the training data, responses were averaged across azimuths before running the regression analysis, since the model could not be fitted for all azimuths and time points separately, as in training sessions one and seven, only half of the items were presented. To compare weights estimated from the training data with weights estimated from the pre- and posttest data, this limited-range regression analysis was applied also to the pre- and posttest data (in addition to the main regression analysis using the full range).

## RESULTS

### Lateralization Responses

To evaluate the overall lateralization performance in our experiment, we calculated a basic, widely used measure, the root-mean-square error (RMSE) between response and stimulus azimuth for the consistent-cue items. The RMSE contains errors due to both systematic bias and response variability. In the pretest, the mean across all participants was 13.04° (*SD* = 4.94). Using a head pointing method in a virtual environment including individual HRTF filtering, Middlebrooks (1999) reported a mean lateral RMSE of 14.5° (*SD* = 2.2) and Majdak et al. (2010) a mean lateral RMSE of 14.4° for untrained participants. Note that the sources in these studies also varied in elevation. However, restricting the range of polar source angles in Majdak et al. (2010), solely including sources close to the horizontal plane, only marginally decreased the lateral RMSE. We assume a similar dependency for the data of Middlebrooks (1999). Our lateralization method using binaural stimuli without HRTF filtering therefore shows comparable accuracy to virtual acoustics studies using a similar response paradigm.

**Fig. 3:**
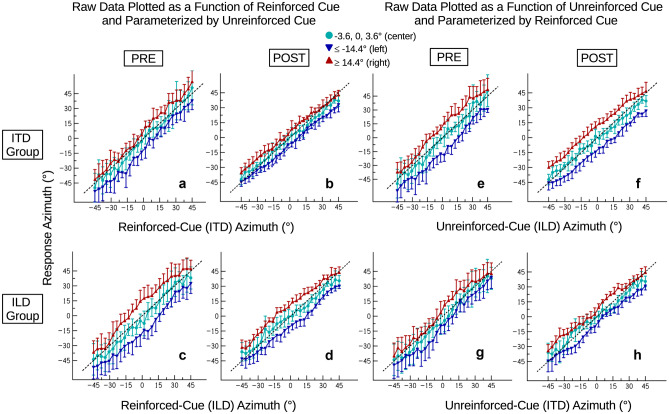
An overview of mean response azimuths across participants as a function of either reinforced-cue azimuth (ITD azimuth for the ITD group, ILD azimuth for the ILD group; panels **a**–**d**, left-hand side) or unreinforced-cue azimuth (ILD azimuth for ITD group, ITD azimuth for ILD group; panels **e**–**h**, right-hand side). The data are parameterized by the offset of the cue not shown on the *x*-axis from the cue shown on the *x*-axis, pooling across three offset ranges: central offsets (−3.6°, 0°, and 3.6°, cyan circles), leftward offsets (≤ 14.4°, blue downward-pointing triangles) or rightward offsets (≥ 14.4°, red upward-pointing triangles). In each panel pair, the panel on the left shows the pretest and the panel on the right shows the posttest. Error bars indicate standard deviations

Figure 3 provides a descriptive overview of the overall results pattern. Statistical analyses of the data are provided in later sections. It plots the mean response azimuth across participants as a function of either the reinforced-cue azimuth (panels a–d, left-hand side) or the unreinforced-cue azimuth (panels e–h, right-hand side). The data are parameterized by the offset of one cue (the cue that is not shown on the *x*-axis) from the other cue (the cue that is shown on the *x*-axis), pooling across three offset ranges: central offsets (−3.6°, 0°, and 3.6°, cyan circles), leftward offsets (≤ 14.4°, blue downward-pointing triangles) or rightward offsets (≥ 14.4°, red upward-pointing triangles). The cyan response curves (showing consistent-cue conditions and conditions with very small cue disparities) are fairly linear and close to the diagonal, showing that participants were able to extract the binaural cues and responded accurately using the employed setup. The separation of the three lines in each panel shows that both cues contributed to the perceived azimuth (e.g., response azimuths are further right for rightward offsets compared to central offsets, independent of which cue is parameterized as shown by the red lines falling above the cyan lines). In the posttest, response slopes were generally shallower compared to the pretest, suggesting an overall compression of response azimuths (reduced range of response azimuths relative to presented azimuths) which is further explored below. The distance between the three curves is indicative of binaural cue weighting. It is larger in conditions where the ITD offset is shown as the parameter (panels c–f), suggesting a larger ITD weight in both groups, which is particularly prominent in the pretest data (panel c versus a and panel e versus g). For panels showing the unreinforced-cue offset as the parameter (a-d), the distance between curves is expected to be smaller in the post- compared to the pretest, assuming a reduction of the unreinforced-cue weight and thus an increase of the reinforced-cue weight. This indeed seems to be the case for both groups (mean vertical separation between downward and upward triangles decreases from 16.1 to 11.6° for the ITD group and from 23.6 to 18.3° for the ILD group). Note, however, that compression also contributes to a reduced distance between curves. In contrast, for panels showing the reinforced-cue offset as the parameter (e–h), binaural cue reweighting would result in increasing distance between curves from pre- to posttest. In this case, however, compression counteracts cue reweighting. Consistent with these assumptions, the distance between curves appears to be more similar in pre- and posttest (mean vertical separation changes only marginally from 23.8 to 22.1° for the ITD group and even increases slightly from 13.2 to 14.5° for the ILD group).

### Binaural-Cue Weights

**Fig. 4:**
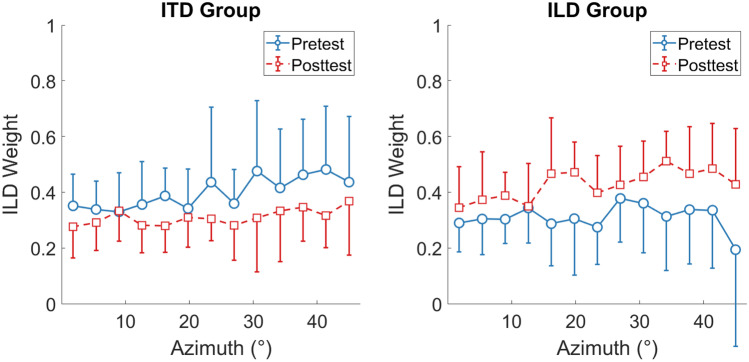
ILD weights as a function of azimuth (after collapsing data across left and right azimuths) derived using a regression analysis. Blue circles show the pretest results and red squares the posttest results, separately for the ITD and ILD groups (individual panels). Note that, by definition, ITD weight = 1 −ILD weight. Error bars show standard deviations

**Fig. 5:**
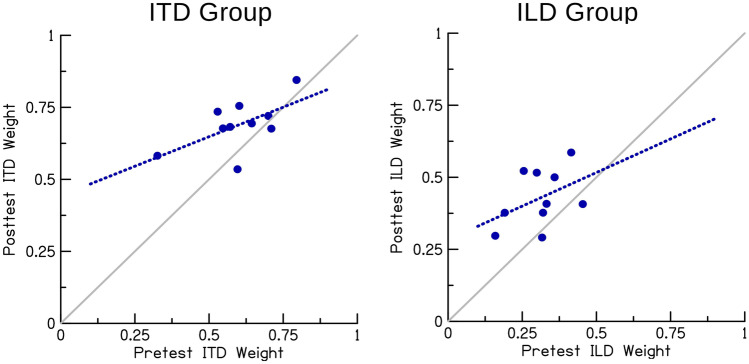
Posttest reinforced-cue weight as a function of pretest reinforced-cue weight including the slopes of linear regressions fitted to the data. The symbols show individual participants

Figure 4 shows the ILD weights (${w}_{ILD}$) as determined by the regression analysis at each azimuth separately for the two groups. Posttest ILD weights (red squares) *decreased* for the ITD group and *increased* for the ILD group compared to pretest ILD weights (blue circles). A 2 × 13 × 2 mixed-design ANOVA with the within-participants factors *time* (pre- vs. posttest) and *azimuth* and the between-participants factor *group* (ITD vs. ILD group) yielded no significant main effects, but a significant *time* × *group* interaction (*F*(1,18) = 20.44, *p* < 0.001, *η*_*p*_^*2*^ = 0.532). All other interactions were non-significant. The lack of a significant main effect of *time* was expected, assuming opposing effects of time on the ILD weights for the two groups. Simple main effect analyses showed that the *time* × *group* interaction was driven by a significant difference between the pre- and posttest in the ITD group (*F*(1,18) = 7.44, *p* = 0.028, *η*_*p*_^*2*^ = 0.293, Bonferroni-corrected), with larger ILD weights in the pretest (*M* = 0.40, *SD* = 0.13) compared to the posttest (*M* = 0.31, *SD* = 0.09), as well as a significant difference between the pre- and posttest in the ILD group (*F*(1,18) = 13.43, *p* = 0.004, *η*_*p*_^*2*^ = 0.427, Bonferroni-corrected), with smaller ILD weights in the pre- (*M* = 0.31, *SD* = 0.09) compared to the posttest (*M* = 0.43, *SD* = 0.10). They further showed that the groups did not differ significantly in the pretest (*F*(1,18) = 3.17, *p* = 0.184, *η*_*p*_^*2*^ = 0.150, Bonferroni-corrected), but there was a significant group difference in the posttest (*F*(1,18) = 8.04, *p* = 0.022, *η*_*p*_^*2*^ = 0.309, Bonferroni-corrected). Figure 5 plots the reinforced-cue weights averaged across azimuths of each participant. Consistent with the significant pre- vs posttest difference in both groups, the weight given to the reinforced cue increased from pre- to posttest for 16 out of 20 participants. Note that the estimates of ${w}_{ILD}$ are independent of response compression under the assumption that compression affects both cues equally, so its effect on the slopes *k*_*ITD*_ and *k*_*ILD*_ cancels out in the final weight estimation (Eq. 1). This assumption was tested using a modeling approach, which yielded a better account of the data for a model version assuming reweighting combined with cue-independent compression than a model version assuming cue-specific compression of the unreinforced cue (see Appendix for details). Taken together, these results suggest that the training induced an azimuth-independent increase in the reinforced-cue weights for both groups.

Next, we addressed the potential implication of apparently asymmetric binaural cue weights (i.e., an overall stronger weighting of ITD cues). Since azimuth had no significant effect on the ILD weights, the weights were averaged across azimuths. The mean ILD weights were significantly smaller than 0.5 (a weight of 0.5 means equal weighting of the two binaural cues) in both the pretest (*M* = 0.35, *SD* = 0.12, *T*(19) = −5.59, *p* < 0.001, *d*_*z*_ = −1.25) and the posttest (*M* = 0.37, *SD* = 0.11, *T*(19) = −5.37, *p* < 0.001, *d*_*z*_ = −1.20), showing an overall dominance of ITD cues. Because a higher baseline (pretest) weight for the reinforced cue potentially limits the room for training-induced reweighting towards the reinforced cue, we first checked if the amount of reweighting, quantified as the post- versus pretest difference in reinforced-cue weights, differed between groups. The amount of reweighting (ITD group 0.09; ILD group 0.12) did not differ significantly between groups (*T*(18) = −0.66, *p* = 0.516, *d* = −0.31), providing no evidence that such ceiling effects might have affected the ITD group. As the distribution of pre- and posttest weights across participants might provide further hints, we examined the posttest reinforced-cue weight as a function of the pretest reinforced-cue weight (see Fig. 5). For the ITD group (left panel), the data points accumulate more towards the upper right quadrant of the plot and the pattern appears to be shallower than the diagonal. Accordingly, the 95 % confidence interval (CI) for the slope of a linear regression fitted to the data did not include 1 (slope 0.41; 95 % CI = −0.03, 0.85). We therefore cannot completely rule out that participants with a high pretest ITD weight were somewhat affected by ceiling effects. For the ILD group (right panel), the data accumulate more toward the lower left quadrant of the plot and the pattern is, compared to the ITD group, more parallel to the diagonal. Consistently, the 95 % CI for the slope of a linear regression fitted to the data did include 1 (slope 0.47; 95 % CI = −0.34, 1.27). Thus, there is no hint for ceiling effects in the ILD group, which is expected given the low pretest ILD weight of all participants.

### Time Course of Cue Reweighting

**Fig. 6:**
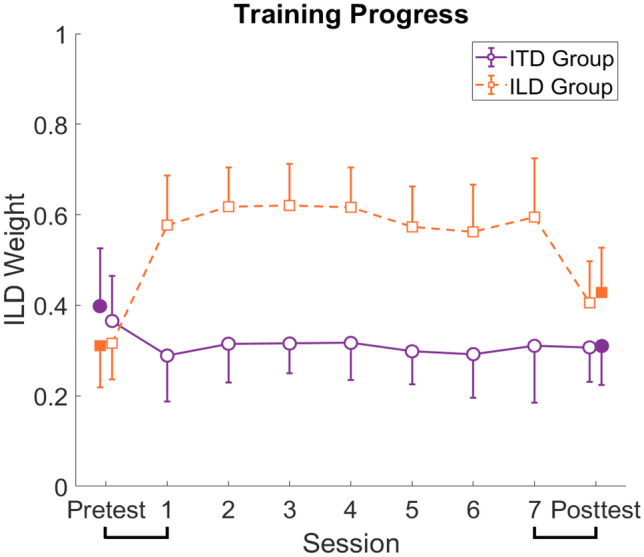
Training progress. ILD weights calculated by limiting the combinations considered for azimuths more lateral than 19.8° to ensure balanced distributions are shown for the pretest, the seven training sessions and the posttest. Purple circles show the results for the ITD group, orange squares for the ILD group. For comparison, the pre- and posttest weights calculated using all data are shown as separate symbols at the far left and far right of the figure. Error bars show standard deviations

We further sought to investigate the time course of cue reweighting across training sessions. Figure 6 shows the training progress for a data subset (see “Methods” section for details) for the two groups. For comparison, the pre- and posttest weights calculated using all data, replotted from the means across azimuth in Fig. 4, are added as separate filled symbols at the far left and far right of the figure. For both groups, the training effect seems to have been induced within the first training session and there appears to be no further change over the course of training. In the ITD group, the relatively small effect appears to have persisted after training, while in the ILD group the initially larger effect appears to have partly dissipated in the posttest. Two repeated-measures ANOVAs (separate for the groups) using the seven training sessions as the within-subjects factor confirmed that there were no significant differences across training sessions. We therefore averaged across training sessions to further explore the relationship between the pre-/posttest and the training. We ran two repeated-measures ANOVAs (one for each group) with the within-subjects factor *time* (pretest–averaged training sessions–posttest) including post-hoc pairwise comparisons. For the ITD group, the main effect of *time* failed to reach significance after correcting for a sphericity violation (*F*(1.27,11.44) = 3.72, *p* = 0.072, *η*_*p*_^*2*^ = 0.292, Greenhouse–Geisser-corrected). The post-hoc pairwise comparisons also did not yield any significant differences (all *p* > 0.191, Bonferroni-corrected). For the ILD group, the repeated-measures ANOVA yielded a significant main effect of *time* (*F*(1,18) = 19.55, *p* < 0.001, *η*_*p*_^*2*^ = 0.685). The post hoc pairwise comparisons showed significant differences between the pretest and the training (*p* = 0.001, Bonferroni-corrected) with smaller ILD weights in the pretest (*M* = 0.315, *SD* = 0.079) compared to the training (*M* = 0.594, *SD* = 0.089), the training and the posttest (*p* = 0.022, Bonferroni-corrected) with larger ILD weights in the training compared to the posttest (*M* = 0.405, *SD* = 092) as well as the pre- and the posttest (*p* = 0.048, Bonferroni-corrected) with smaller ILD weights in the pretest compared to the posttest. Hence, the ITD group showed a modest, albeit non-significant decrease in the ILD weight from the pretest to the first training session and no change afterwards. In contrast, the ILD group showed a larger, significant increase in the ILD weight during the first training session and a significant reduction of the ILD weight from the last training session to the posttest. Note that these training results should be interpreted with caution, given that only a subset of data could be analyzed, given the requirement to estimate binaural cue weights independent of response bias.

### Response Compression

**Fig. 7:**
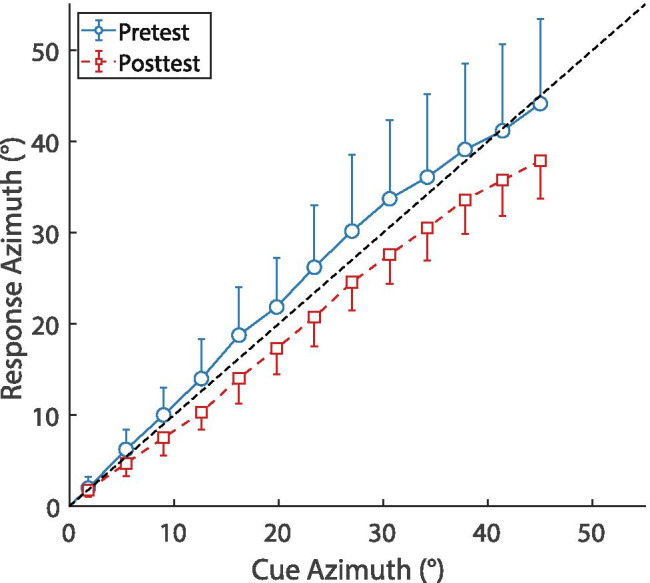
Estimated response azimuths for the consistent items (i.e., the parameter Q from the regression analysis) as a function of cue azimuth, pooled across the groups. Blue circles show the pretest results and red squares the posttest results. Error bars show standard deviations

Next, we examined the response compression (i.e., overall shallower lateralization slopes in the posttest) from pre- to posttest observed for both groups (see description of Fig. 3). Figure 7 shows the estimated response azimuths for consistent cues based on the regression analysis (i.e., the estimated values of *Q* from Eq. 1), pooled across groups. Lateralization in the pretest was fairly accurate, since the estimated response azimuths are similar to the cue azimuths (i.e., close to the diagonal) and pretest slopes of estimated response vs. cue azimuths were not significantly different from 1 (*M* = 1.04, *SD* = 0.25, *T*(19) = 0.81, *p* = 0.431, *d*_*z*_ = 0.18). Posttest slopes, however, were shallower than pretest slopes and significantly different from 1 (*M* = 0.87, *SD* = 0.10, *T*(19) = −5.90, *p* < 0.001, *d*_*z*_ = −1.32), suggesting a systematic and apparently linear compression of responses. Subjecting the ratio of estimated responses/cue azimuths to a 2 *(time)* × 13 *(azimuth)* × 2 (*group*) mixed-design ANOVA yielded a significant main effect of *time* (*F*(1,18) = 18.19, *p* < 0.001, *η*_*p*_^*2*^ = 0.503), but neither the main effects (*azimuth* and *group*) nor the interactions were significant, suggesting that this compression of responses from pre- to posttest is indeed linear and similar in both groups.

### Change in Lateralization Precision and Split-Image Perception

**Fig. 8:**
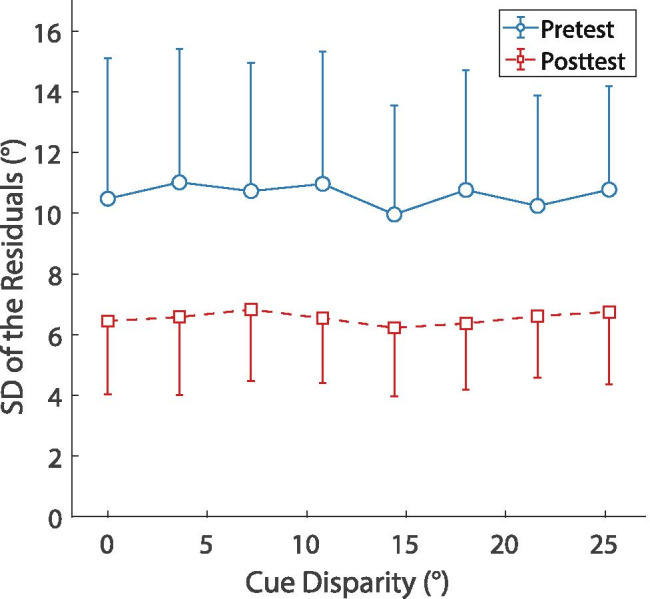
Response variability (defined here as the standard deviation of the residuals of the regression analysis) as a function of cue disparity in the pre- and posttest pooled across groups. Blue circles show the pretest results and red squares the posttest results. Error bars show standard deviations

Finally, we attempted to quantify the training-induced change in overall lateralization precision (i.e., the consistency in lateralization responses; see Heffner and Heffner 2005) and confirm that the participants did not perceive split images (Hafter and Jeffress 1968) when stimuli with inconsistent cues were presented. To that end, response variability was calculated by computing the residuals (where, for each response, the residual is defined as the deviation of the actual response azimuth from the response azimuth predicted by the regression analysis from Eq. (1) and then computing the standard deviation of these residuals. Figure 8 shows the results as a function of cue disparity, averaged across groups. The response variability was systematically lower in the posttest than in the pretest, while there seems to be no systematic effect of cue disparity. The mean variability across groups and all binaural cue disparities decreased from 10.62 (*SD* = 3.90) in the pretest to 6.54 (*SD* = 2.17) in the posttest. A 2 (*time)* × 8 (*cue disparity)* × 2 (*group)* mixed-design ANOVA showed significant main effects of the factors *time* (*F*(1,18) = 27.24, *p* < 0.001, *η*_*p*_^*2*^ = 0.602) and *cue disparity* (*F*(4.18,75.26) = 2.51, *p* = 0.046, *η*_*p*_^*2*^ = 123, Greenhouse–Geisser-corrected) but no significant effect of the factor *group* and no significant interactions. Post hoc pairwise comparisons revealed that the main effect of *cue disparity* was driven by a significant difference between cue disparities of 14.4° and 25.2° (*p* = 0.008, Bonferroni-corrected). However, variability did not systematically increase with increasing cue disparity nor adopt an inverted u-shape. Either of these two patterns might be expected if larger cue disparities had evoked the perception of split images. As a further check for the possibility of split image perception, we inspected the response distributions as a function of binaural cue disparity for each participant and found no systematic indications for distributions being bimodal or centered close to one cue azimuth only, as may be expected in the case of split image perception. Thus, these results provide no indication for split image perception and instead suggest that participants perceived a single compact auditory image for all cue disparities included in this study.

## DISCUSSION

This study is, to our knowledge, the first one to provide evidence that it is possible to selectively increase the relative weighting of either ITD or ILD cues. This binaural-cue reweighting was induced through lateralization training in a virtual audio-visual environment, employing visual reinforcement as well as symmetric azimuthal variation of unreinforced cues around reinforced cues. The results demonstrate that the auditory system flexibly adjusts the contribution of each of the two binaural cues to the perceived lateral position of an auditory object based on previous experience.

### Factors Contributing to Binaural-Cue Reweighting

While several studies have addressed the plasticity of the spatial auditory system to spatial cue modifications (e.g., Kumpik et al. 2010; Shinn-Cunningham et al. 1998), the only two published studies we are aware of explicitly addressing binaural-cue reweighting (Jeffress and McFadden 1971; Kumpik et al. 2019) produced inconclusive results. Jeffress and McFadden observed no reweighting effect and while Kumpik et al. report an increase in ILD weighting after ITDs were randomized, they did not observe an increase in ITD weighting when ILDs were randomized and observed an even stronger increase in ILD weighting when spatially consistent ITDs and ILDs were presented. These different results (both regarding previous literature as well as the present study) can likely be attributed to differences in the methodology, as discussed in the introduction, suggesting that factors necessary to induce binaural-cue reweighting may include active listening, intuitive responding, bottom-up multisensory integration, and auditory stimuli that contain salient ITDs as well as salient ILDs.

Our study employed five key manipulations during training to maximize chances for inducing binaural-cue reweighting: (1) presenting visual stimuli at the reinforced-cue azimuth after the response as top-down feedback, (2) presenting visual stimuli simultaneously with the second sound presentation to tap into multisensory bottom-up processes, (3) requiring responses via head-turn, therefore also involving proprioceptive information, (4) using narrow-band noise at a frequency range for which neither ITDs nor ILDs are known to dominate, and (5) manipulating the stability of the cues by varying the unreinforced cue over a larger range than the reinforced cue. Trapeau and Schönwiesner (2015) observed that, when participants wear earplugs consistently delaying the sound at one ear, they remap their auditory space based on that delay instead of increasing the weight given to preserved ILD cues. Therefore, cue variation as opposed to a predictable cue manipulation might also be an important factor contributing to cue reweighting.

### Time Course of Cue Reweighting

Although our data are not optimally suited for studying the time course of reweighting (e.g., not all ITD/ILD combinations included in the pre- and posttest were included in the training), the current analysis of the training data suggests that reweighting occurred predominantly within the first training session. This is consistent with Kumpik et al. (2019), who reported binaural cue reweighting to occur within less than 1 h of training. Kumpik et al. (2010) on the other hand did not observe adaptation (reweighting towards monaural cues in this case) when all training trials were performed in one day, but rather found continuous improvement across all 7 or 8 training days. A plausible explanation could be that the participants in their study needed to learn to exploit monaural spectral cues for azimuthal localization when the binaural cues were disrupted, while such learning was not required in our study given that both binaural cues are used by default.

It should also be noted that the time course seems to differ between the two groups in the current study. While the ITD group showed less reweighting from the pretest to the training which then remained stable through the posttest, the ILD group showed stronger reweighting from the pretest to the training, part of which then got lost from the last training session to the posttest. Wright and Fitzgerald (2001) also reported different time scales for ITD vs. ILD discrimination learning. However, these time scales differ from the current observations. Namely, they report an initial rapid improvement for both cues that generalizes across conditions followed by a slower improvement for ILD discrimination only. Another possibility to consider is that the adaptation occurs on multiple time scales, as, for example, observed in the ventriloquism aftereffect (Bosen et al. 2018). Specifically, the reweighting of the ILD group might consist of a quick strong component that is spontaneously reversed when the visual feedback stops, combined with a weaker, also quick, but more sustained reweighting. In comparison, the ITD group might only show the more sustained component. This would be interesting to explore in future studies.

### Conscious vs. Unconscious Reweighting

An important question is whether the current results could have been mediated by conscious or strategic listening. We consider strategic reweighting very unlikely given the myriad of reinforced- and unreinforced-cue azimuths spread across a wide range. Moreover, the disparity between ITD and ILD azimuths was intentionally limited (maximum of 25.2°) with the goal to avoid the perception of split images (e.g., Hafter and Jeffress 1968) which could theoretically allow for strategic listening, if the two binaural cues could be distinguished. In fact, all participants (as well as the authors during informal piloting) reported perceiving compact auditory images, with no indication of split images. Consistent with these subjective reports, we observed similar response variability across cue disparities and no indications for response distributions being bimodal or centered close to the azimuth of one binaural cue only. The observation that there was no improvement after the first training session (especially in the ILD group whose ILD weights were still far from 1) further makes conscious reweighting unlikely. Taken together, these results suggest that binaural-cue reweighting occurs at a more low-level, unconscious processing stage.

### Response Compression

We found a systematic compression of the response azimuth range from pre- to posttest. This can at least partly be attributed to limiting the azimuth range of the reinforced cue to ± 45° during training. While the unreinforced cues ranged up to ± 70.2° and auditory stimuli were certainly perceived beyond ± 45°, reinforced-cue azimuths and therefore also the visual reinforcement were restricted to ± 45°, likely triggering a mapping to this azimuth range. Interestingly, the resulting response compression occurred not only at the edges, but across the entire azimuth range, and followed a linear function. This result is consistent with an earlier study showing that various nonlinear mapping functions between auditory and visual azimuth space resulted in response azimuths following a linear approximation of these functions (Shinn-Cunningham et al. 1998). Furthermore, fatigue or decreased willingness by the participants to exploit the whole azimuthal response range in the posttest might have contributed to response compression.

We considered the possibility that response compression was specific to the unreinforced cue, given that it was varied over a larger range than the reinforced cue during training. However, the results of a simple modelling approach indicate that the compression is cue-independent, suggesting that it occurs after the information of the two binaural cues is integrated (see Appendix).

### Pretest ITD Weights

The dominance of ITD cues observed in the present study is consistent with the literature (Macpherson and Middlebrook 2002; Wightman and Kistler 1992). Macpherson and Middlebrooks observed that for their wideband stimulus condition, participants either weighted ITDs and ILDs equally or weighted ITDs more. Since our passband lies in between Macpherson and Middlebrooks’ low-pass and high-pass stimulus conditions and some low- and high-frequency energy was present due to the roll-off of our band-pass filter, Macpherson and Middlebrooks’ wideband stimuli appear most comparable to the stimuli used in the present study.

### Implications of the Particular Auditory Stimuli Used

The passband of our stimuli lies in a frequency region where ITDs are conveyed only via the temporal envelope. Bernstein and Trahiotis (1982), however, observed that residual energy below the stimulus passband, comparable to our study, can provide access to salient low-frequency (likely fine-structure based) ITD cues. Some influence of fine-structure ITDs in the present study is in fact suggested by the overall stronger weighting for ITDs, like in Macpherson and Middlebrooks’ (2002) wideband condition.

We intentionally chose a frequency range for our stimuli that lies in between typically ITD- or ILD-dominant regions so that both ITD and ILD weighting had the potential to be increased. Future studies may investigate whether reweighting also occurs for more natural broadband stimuli including low- and high-frequency regions where ITDs and ILDs, respectively, are known to dominate perceptually. Additionally, it is unclear whether the training-induced reweighting generalizes to non-trained frequency regions. Wright and Fitzgerald (2001), for example, observed different generalization patterns of ITD and ILD sensitivity training.

Furthermore, we presented auditory stimuli via headphones using constant binaural cues across the stimulus spectrum rather than HRTF filtering. This was done to ensure that the stimuli did not convey monaural spectral localization cues that are potentially informative about the azimuth of the stimulus, to reveal purely binaural-cue reweighting. Kumpik et al. (2010), for example, observed an increased weighting of monaural cues and no adaptation to changed binaural cues when monaural spectral cues were preserved at one ear. As monaural and binaural localization cues interact in everyday life, the effect of binaural-cue reweighting on more realistic stimuli is an interesting topic for future studies.

### Applications

Binaural-cue reweighting might be useful for situations in which one of the cues is particularly informative while the other cue is less reliable or even misleading. For example, since listeners benefit from the presence of both ILD and ITD cues in spatial release from speech masking (e.g., Ellinger et al. 2017; Kidd et al. 2010), binaural-cue reweighting may be a means to cope with the changing reliability of binaural cues in speech in multi-talker or differing acoustically complex environments. Consistent with this suggestion, Rakerd and Hartmann (2010) observed that ILDs were increasingly favored as the binaural waveform coherence decreased after introducing reverberation.

Similarly, our results might have implications for listeners with cochlear implants (CIs). It has been shown that localization in the horizontal plane with current CI systems is almost entirely based on ILDs, while ITDs contribute only very little or not at all (Grantham et al. 2008; Seeber and Fastl 2008). On one hand, this is due to the properties of current envelope-based CI systems, which encode no useful ITDs in the pulse carriers and whose envelope ITD cues for real-life stimuli are not very salient (Grantham et al. 2008; Laback et al. 2004, 2011). On the other hand, even when presenting pulse carrier ITDs highly controlled via a research system at a single interaural electrode pair, CI listeners’ sensitivity is greatly reduced and much more variable across listeners compared to normal-hearing listeners’ carrier ITD sensitivity (Laback et al. 2007; Majdak et al. 2006; van Hoesel 2007). Several explanations have been proposed for this perceptual deficit in electric hearing (see, e.g., Laback et al. 2015). Considering our current results, it might partly be a result of reweighting of the binaural cues over time. Specifically, it is possible that binaural cue reweighting takes place after CI implantation, resulting in a stronger weighting of the ILDs which consistently indicate sound source locations, and a decreased weighting of the ITDs which are not reliably and saliently provided by the CI listeners’ clinical devices.

### Summary and Overall Conclusions

In conclusion, our results suggest that reweighting of auditory localization cues is not limited to monaural vs. binaural cues, which has been shown in previous studies, but that reweighting of the two binaural cues ITDs and ILDs is also possible. Specifically, we show that the weighting of both ITDs and ILDs can be selectively increased by reinforcing the respective cue through lateralization training. This could play a role in adapting to variable acoustic environments, be a factor contributing to the low contribution of ITDs to sound localization in CI listeners, and have potential applications, for example, in training for unfamiliar audio-visual environments or with hearing devices that impede one of the two binaural cues.

## Appendix

The estimate of ${w}_{ILD}$ is independent of response compression under the assumption that compression affects both cues equally, so that its effect on the slopes *k*_*ITD*_ and *k*_*ILD*_ cancels out in the final weight estimation (Eq. 1). However, as unreinforced-cue azimuths were varied over a wider range (± 70.2°) than reinforced-cue azimuths (± 45°) during training, it is conceivable that the training led to a specific compression of the unreinforced cue while the reinforced cue was not affected by compression. Such a scenario could be an alternative interpretation of the results because it would reduce the relative contribution of the unreinforced cue, similar to the effect of cue reweighting. In this Appendix, we describe the results of a simple modeling approach to determine which of these two scenarios quantitatively better characterizes the current data. To that end, we predicted lateralization responses based on weighted averages of the cue azimuths under different compression assumptions and compared those predictions to the actual posttest data. The mean posttest data of each group from Fig. 3 were used, for which either the unreinforced cue was varied around the reinforced cue (panels b and d) or the reinforced cue was varied around the unreinforced cue (panels f and h), categorized by cue offsets “center,” “left,” or “right” (see caption of Fig. 3). Predictions were made separately for each azimuth.

For a given condition with a certain combination of binaural-cue values, corresponding cue azimuths were first multiplied by a compression factor, C (C = 1: no compression: C < 1: compression), which was either the same (cue-independent) or different (cue-specific) for the two cues (see model versions below). The resulting values where then subjected to weighted averages as determined by the parameter ${w}_{ILD}$ (see description of regression analysis in the “Methods” section) to form the predicted response azimuth. Depending on the model version, either or both parameters C and ${w}_{ILD}$ were freely varied (in steps of 0.01) and the parameter(s) resulting in the highest prediction accuracy were determined. Prediction accuracy was determined by means of the RMS deviation between observed and predicted response azimuths (referred to as RMSE). All conditions fulfilled the requirements for convex optimization.

We compared the performance of two model versions. In the *cue-specific-compression* (*CSC*) model, C was freely varied for the unreinforced cue while it was fixed for the reinforced cue. Specifically, for the reinforced cue, C was based on the consistent-cue pretest data (1.070 and 1.024 for the ITD and ILD groups, respectively). Parameter ${w}_{ILD}$ was fixed and taken from the regression analysis of the pretest data, where C is very close to 1 and, thus, weight estimation is very unlikely to be confounded by cue-specific compression. In the *reweighting-and-cue-independent-compression* (*RCIC*) model, both C and ${w}_{ILD}$ were freely varied, i.e., all combinations of the two parameters were evaluated.

The results are summarized in Table 2. The RCIC model resulted in a lower RMSE (1.09°) than the CSC model (1.54°). This difference was significant for both the ITD group (*T*(12) = 3.57, *p* = .004, *d*_*z*_ = 0.99) and the ILD group (*T*(12) = 3.09, *p* = .006, *d*_*z*_ = 0.86) when comparing the means across azimuths. Moreover, the estimates of ${w}_{ILD}$ across azimuths by the RCIC model correspond very well to the respective posttest estimates by the regression analysis (*r*^*2*^ = 0.9 and 0.94 for the ITD and ILD groups, respectively). Also, mean C estimates by the RCIC model (0.89 and 0.87 for ITD and ILD groups, respectively) correspond very well to the bias estimates of the regression model (0.87 and 0.87 for ITD and ILD groups, respectively). In summary, the model results suggest that the training effect observed in the posttest was more likely induced by cue reweighting combined with cue-independent compression than by cue-specific compression of the unreinforced cue.

**Table 2:** Results of modeling the posttest data for the ITD group (upper two rows) and ILD group (lower two rows) using two model versions, the cue-specific compression (CSC) model and the reweighting and cue-independent compression (RCIC) model. RMSE refers to the RMS deviation between observed and predicted response azimuths, C to the compression factor, and ${w}_{ILD}$ to the ILD weight

Model version	Compression	Cue weight	Mean RMSE across azimuths	SD	C	SD	*W*_*ILD*_	SD
Cue-specific compression (CSC)	Varied	Pretest	1.54	0.55	0.70	0.10	-	-
Reweighting and cue-independent compression (RCIC)	Varied	Varied	1.09	0.36	0.89	0.03	0.35	0.03
Cue-specific compression (CSC)	Varied	Fixed (pretest)	2.37	0.85	0.79	0.03	-	-
Reweighting and cue-independent compression (RCIC)	Varied	Varied	1.96	0.60	0.87	0.03	0.45	0.06
